# 1,2-Naphthoquinone-4-sulfonic acid salts in organic synthesis

**DOI:** 10.3762/bjoc.18.5

**Published:** 2022-01-05

**Authors:** Ruan Carlos B Ribeiro, Patricia G Ferreira, Amanda de A Borges, Luana da S M Forezi, Fernando de Carvalho da Silva, Vitor F Ferreira

**Affiliations:** 1 Universidade Federal Fluminense, Departamento de Química Orgânica, Instituto de Química, Campus do Valonguinho, 24020-150, Niterói-RJ, Brazil; 2 Universidade Federal Fluminense, Faculdade de Farmácia, Departamento de Tecnologia Farmacêutica, 24241-000, Niterói-RJ, Brazil

**Keywords:** biological activities, derivatization reagents, β-NQS, organic synthesis

## Abstract

Several low molecular weight naphthoquinones are very useful in organic synthesis. These compounds have given rise to thousands of other naphthoquinones that have been tested against various microorganisms and pharmacological targets, including being used in the preparation of several drugs that are on the pharmaceutical market. Among these naphthoquinones, the series of compounds prepared from 1,2-naphthoquinone-4-sulfonic acid salts (β-NQS) stands out. In addition to being used in organic synthesis, they are excellent analytical derivatization reagents to spectrophotometrically determine drugs containing primary and secondary amino groups. This review summarizes the literature involving β-NQS.

## Introduction

The general class of quinones is very important because their compounds show biological activities against several pathogens related to important diseases and are used for the production of special materials [1–4]. These compounds are biosynthesized by oxidative processes of catecholamines and other compounds, but they can also be ingested as exogenous products of air and water. The most common quinones, such as benzoquinone, naphthoquinone, anthraquinone, and phenanthrenequinone, can be formed by incomplete combustion or photooxidation of their respective polycyclic aromatic hydrocarbons (PAHs) [5–6].

**Figure 1:**
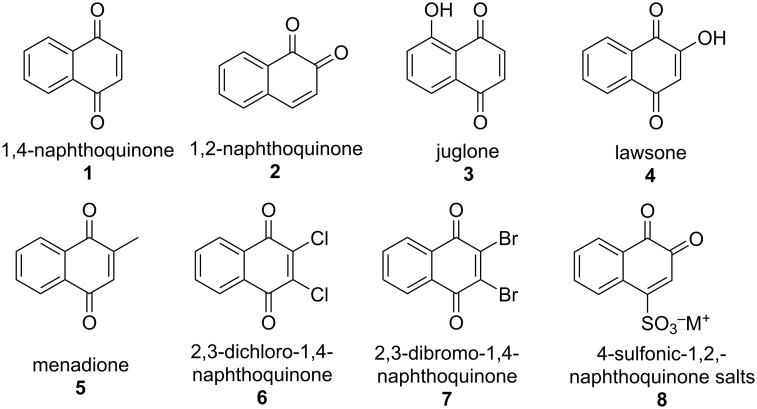
Naphthoquinones are commonly used in organic synthesis.

Among all of the compounds in this class, 1,2- and 1,4-naphthoquinones stand out, as they are present in plants, fungi, lichens, bacteria, algae, viruses, insects, and higher organisms and perform several biochemical functions, such as defense, transference of electrons in various oxidative processes in aerobic metabolism, photosynthesis, oxidative phosphorylation, blood clotting, and other electron transport reactions [7–8]. These biochemical functions give them several biological activities, such as antibacterial, fungicidal, antimalarial, trypanocidal, and antitumor. For this reason, many plant extracts that are rich in naphthoquinones continue to be widely used in folk medicine in several countries. More than 350 naphthoquinones that have been isolated from nature are described in the literature, and it is the most abundant structural subunit in the quinone family. Through the synthesis of new naphthoquinones by innovative methods, this class is constantly expanding. Several hypotheses have been formulated and tested to explain the biological activity of these substances. In general, activities against microorganisms are related to the ability to accept one and/or two electrons through a redox cycle promoted by the 1,2- or 1,4-naphthoquinone system. In this cycle, transient reactive oxygen (ROS) and nitrogen (RNS) species are formed as free radicals, peroxides, superoxide anions, radical anions, or dianions. These species generated inside cells accelerate hypoxia and cause several damages to its components, such as carbohydrates, lipids, membrane components, and enzymes that are critical for DNA replication [9–12]. Most synthetic strategies toward naphthoquinones with potential biological activity start from natural and synthetic naphthoquinones, inserting new fragments in the general structure or modifying functional groups. In Figure 1, eight low molecular weight naphthoquinones are highlighted, which are the most commonly used in organic synthesis. Among them, quinones **1**–**5** are naturally occurring, are simple to prepare and are commercially available. Others are exclusively synthetic (**6**–**8**) and prepared from naphthoquinones **1** or **2**. Except for naphthoquinone **8** (β-NQS), the applications in organic synthesis of all other in Figure 1 shown naphthoquinones are already summarized in reviews [13–20].

**Figure 2:**
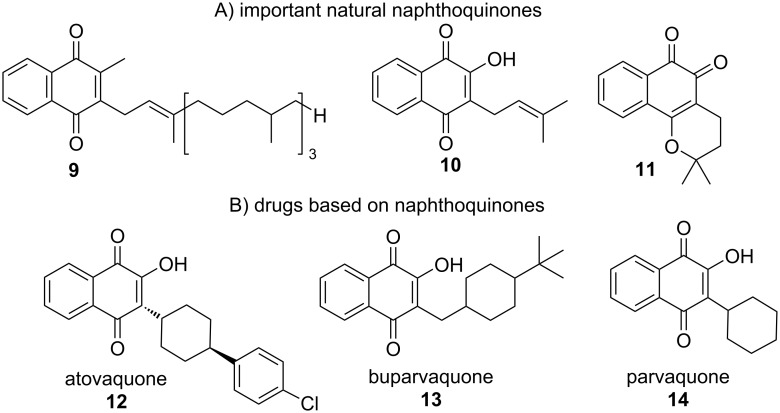
Some important natural and synthetic naphthoquinones.

Several natural naphthoquinones with antibacterial, fungicidal, antimalarial, antiviral, trypanocidal, leishmanicidal, and antitumor activity serve as inspiration for the pharmaceutical industry [21–24]. However, they are considered as Pan Assay Interference compounds (PAINS) because they display biological activity in many assays, but because of such reactivity it can be very difficult to advance to the clinic against drug targets [25–26]. Among the most prominent natural naphthoquinones are vitamin K1 (**9**), lapachol (**10**), and β-lapachone (**11**) (Figure 2A). All of these compounds have important characteristics, but it should be noted that in addition to their biological properties, these compounds have served as raw materials for the synthesis of new naphthoquinone derivatives [27]. Drugs belonging to the class of naphthoquinones are on the pharmaceutical market for the treatment of various diseases. In Figure 2B, three drugs are highlighted that continue to be used in medical practice. Atovaquone (**12**) is a drug that targets the elimination of the parasite *Plasmodium* spp*.* which is the etiological agent of malaria [28–29]. According to data from the World Health Organization (WHO), in 2018, there were approximately 228 million cases of malaria worldwide, with the majority of cases in Africa. It is a serious illness that can lead to death if not treated immediately. This medication has therapeutic use for the treatment or prevention of mild cases of *Plasmodium vivae* infection. Two other drugs structurally similar to lapachol, buparvaquone (**13**) and parvaquone (**14**), are used to treat animal diseases, such as bovine theileriosis (east coast fever, corridor disease, Zimbabwean theileriosis, and tropical theileriosis) [30–33]. Buparvaquone (**13**), phosphate prodrugs, and some formulations were evaluated in vitro and in vivo against *Leishmania donovani*, which causes visceral and cutaneous leishmaniasis. It has been observed that the prodrugs improved efficacy when compared to buparvaquone. Parvaquone (**14**) is a naphthoquinone with antitheilerial properties that is commercialized for the treatment of East Coast fever. This drug is effective in the treatment of cattle infected with Theileria annulata transmitted by the brown tick *Rhipicephalus appendiculatans* [34–36].

As part of our research program on the synthesis of biologically active quinones, we are interested in the synthesis and biological evaluation of naphthoquinones obtained by short routes from readily available starting materials [37–39]. This review summarizes literature data involving 1,2-naphthoquinone-4-sulfonic acid salts (β-NQS), organized based on the general classification of reactions, and explores the possibility of providing practical guidance to synthetic chemists for further research on naphthoquinone compounds.

## Review

### Synthesis of 1,2-naphthoquinone-4-sulfonic acid salts

β-NQS are easily synthesized by traditional methods and commercialized by dozens of chemical companies, such as VladaChem, Ambeed, eNovation Chemicals, Tokyo Chemical Industry, Acros Organics, Abcr GmbH, Sigma-Aldrich, and others. Due to this wide commercial availability, these compounds have been widely used in several industrial applications as analytical reagents for the determination of amines and in the synthesis of other hybrid naphthoquinones [40–42].

**Scheme 1:**
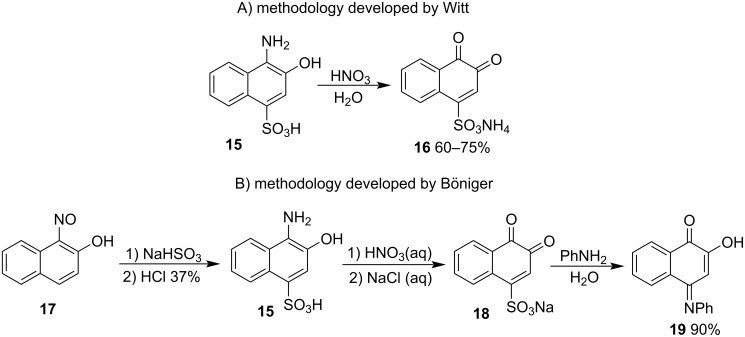
Synthetic studies of BNQs and reactions with amines.

β-NQS preparation methods generally employ β-naphthol or 1-amino-β-naphthol as starting materials. The first procedure was developed by Witt [43] in the late 19th century when he prepared 1,2-naphthoquinone-4-sulfonic acid ammonium salt (**16**) in a 60–75% yield from 1-amino-β-naphthol-4-sulfonic acid (**15**) by oxidation with nitric acid in an aqueous medium (Scheme 1A). In 1894, Böniger developed [44] the first reaction of β-NQS with phenylamines. He synthesized β-NQS using a modified method developed by Witt, which quickly reacted with different amines to form colored products with reddish hues. The reaction with aniline forms the substitution product of the sulfonic group with a phenylamino group in a 90% yield. In his study, he proposed that the structure of the nucleophilic addition product was tautomer **19** (Scheme 1B).

**Scheme 2:**
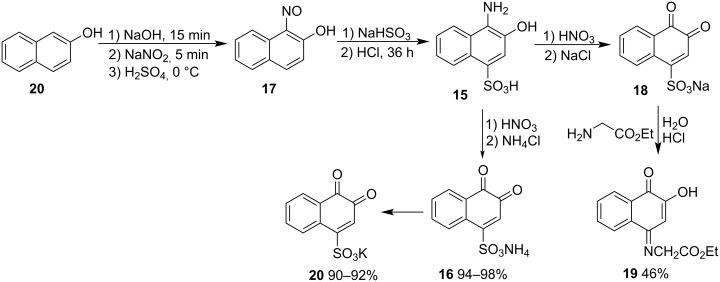
Methods described for the synthesis of β-NQS.

In search of a reagent that could form stable adducts with amino acids to be used as colorimetric indicators, Folin [45] developed a method based on naphthoquinone since there were records in the literature that indicated that naphthoquinones reacted with amines and proteins to form colored products. Among the tested *o*-naphthoquinones, he found that 1,2-naphthoquinone-4-sulfonic acid sodium salt (β-NQSNa, **18**) was very effective as a colorimetric indicator of blood amino acids. This compound came to be called Folin's reagent. To achieve **18** with adequate purity to be used in the tests, an elaborate large-scale synthetic route was developed. β-Naphthol (**20**) was transformed into α-nitroso-β-naphthol (**17**); then, in a single step, a sulfonic group was added, and the nitrous group was reduced, forming compound **15**, which was transformed into β-NQSNa (**18**) after oxidation with nitric acid. Despite not knowing exactly the structure of the adduct, Folin speculated that the reaction probably occurs in the *o*-quinone moiety group. Subsequently, Obo [46] demonstrated that the reaction of β-NQSNa (**18**) and glycine ethyl ester form **19** in a 46% yield, indicating that the reaction occurred at C4 (Scheme 2). Fu and co-workers [47] prepared a new electrochemical sensor for the specific recognition of cholylglycine, which is a combination of cholic acid and glycine. The β-cyclodextrin/graphene oxide composite forms an inclusion complex with a β-NQS guest. The amino group of cholylglycine can bind to β-NQS by a nucleophilic substitution reaction, resulting in a decrease in the electrochemical signal. Danielson [48] found some errors in Folin’s analytical method and optimized it for better amino acid determination. Martin and Fieser [49] described an optimized method, analogous to Folin’s procedures, with temperature control, producing β-NQSNH_4_ **16** and β-NQSK **20** in high yields (Scheme 2).

It is possible to identify three alternatives for the functionalization of β-NQS with amines: a) substitution of sulfonate by secondary amines; b) substitution of sulfonate by primary amines, followed by isomerization; and c) double addition of primary amines. In addition, other nucleophiles can also be used. All cases will be covered throughout the text.

### Synthesis of 4-amino-β-naphthoquinones and analogues from β-NQS

After the first discovery that β**-**NQS reacts quickly with amines to form colored products in good yields, this reagent became quite popular in quantitative analytical determinations of some drugs containing free primary and secondary amino groups [50–51]. As β-NQS is commercially available, it has become a widely used reagent for the chromogenic determination of pharmaceutical amines by spectrophotometry in pharmaceutical formulations [52]. This method, which uses a reagent to form a colored product and determine its concentration by a spectrophotometric method, is the most convenient, simple, and inexpensive method for analytical work. Hiyama [53] noted that little is known about the biological activities of sulfonic naphthalene derivatives, despite being important intermediates for the synthesis of dyes. Then, he prepared several naphthalene sulfonic derivatives and tested them for their effects on bacteria and viruses, but none of the compounds presented important activity.

**Figure 3:**
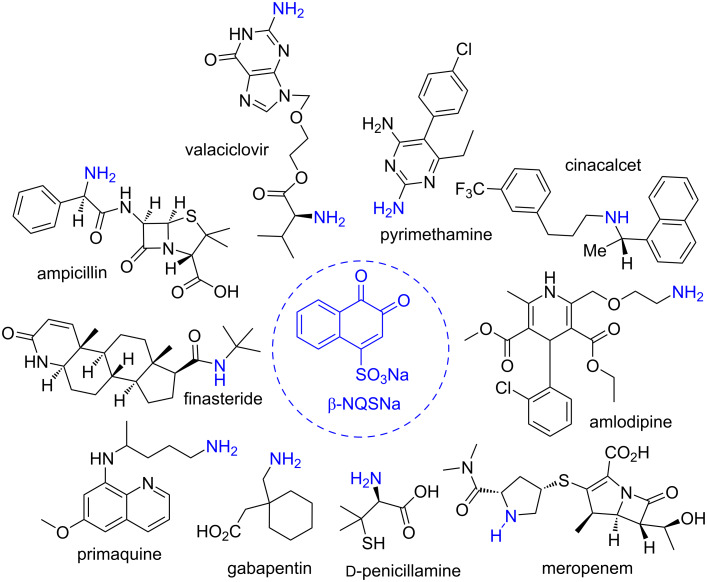
Drugs detected using β-NQSNa.

Hashimoto and co-workers [54] were the first to apply β-NQSNa for the qualitative analysis of phenethylamine derivatives (amphetamine, methamphetamine, 2,5-dimethoxy-4-methylamphetamine, mescaline, ephedrine, and norephedrine). The reaction products were separated by thin-layer chromatography and analyzed by elemental analysis, nuclear magnetic resonance, infrared spectroscopy, and mass spectrometry. In addition, several authors concluded that this was a good method for analytically determining low levels of activated aromatic amines in drugs. This method continued to be used over the years and was subsequently optimized by a spectrophotometric determination technique coupled with continuous flow [55]. In addition to the sulfonic acid substitution reaction of position C4 of β-NQS, quinone can be involved in a redox process and, therefore, can be used as an electrode in electrochemical processes. Subsequently, Legua and co-workers [56–57] applied this method to determine amphetamines in urine. Figure 3 highlights some important drugs containing primary or secondary amino groups in capsules, tablets, powders, formulations, formulations of associated drugs, injection formulations, and biological fluids [42,58–69].

**Scheme 3:**
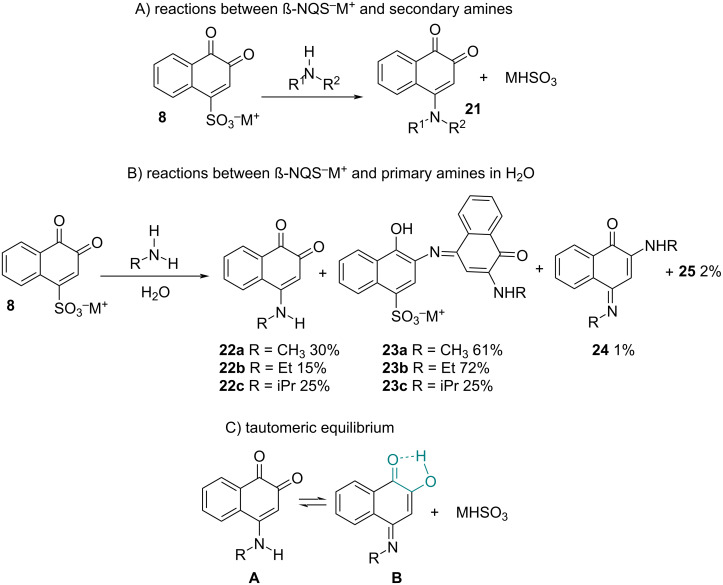
Reactions between β-NQS and amines.

β-NQS reacts with aliphatic and aromatic amines, secondary or primary, by substituting the sulfonic acid group at position C4. These reactions are dependent on the structures of the aliphatic amino reagents. In the case of secondary aliphatic amines, 4-alkyl- (or aryl-) amino-1,2-naphthoquinones **21** are formed (Scheme 3A), but using primary amines forms a product mixture (Scheme 3B), mainly due to a tautomeric equilibrium (Scheme 3C) [41]. Hartke and Lohmann [70] studied the reaction of β-NQS with secondary aliphatic amines in detail and observed that the 4-amino-1,2-naphthoquinone **21** products are yellow. However, reactions with primary aliphatic amines also form 4-amino-1,2-naphthoquinones **22**, but they are violet in color. Structure **22** cannot be responsible for the violet color, and it is attributed to other byproducts. When the reaction with equimolar amounts of β-NQSNa with methyl-, ethyl- and isopropylamine takes place in water at room temperature, a mixture of products is formed, among which **22** and **23** are the majority. It is important to note that the complexity of the reaction of β-NQS with primary aliphatic amines has already been reported in the literature. Fieser and Fieser [71] studied the reduction potentials of various naphthoquinones and reported that they were unable to obtain 4-arylamino-1,2-naphthoquinones from β-NQSNa but that these derivatives can be readily prepared from 4-ethoxy-1,2-naphthoquinone. Similarly, Yano and co-workers [72] studied the tautomeric equilibrium of 4-arylamino-1,2-naphthoquinones in DMSO-*d*_6_, pyridine-*d*_5,_ and NaOD solutions in D_2_O. In neutral solvents, the most stable tautomer is 4-arylamino-1,2-naphthoquinone **A**, while in weakly basic solvents, or ethanolic sulfuric acid, **B** is the most stable tautomer (Scheme 3C).

Reactions employing equimolecular amounts of β-NQS and primary arylamines are cleaner and form the substitution product at position C4. Fieser and Fieser were the first to study tautomerism between 4-arylamino-1,2-naphthoquinone **A** and 2-hydroxy-1,4-naphthoquinone-4-arylimine **B** (Scheme 3C) using the redox potential compared to the pH of the medium.

It was observed that naphthoquinones **A** prevail in all pH regions except for extreme acidity, where there is a shift to the form of 2-hydroxy-1,4-naphthoquinone-4-arylimines [73–74]. However, in weakly acidic or alkaline solutions, **A** is the most stable tautomer (Scheme 3C) [75]. Fragoso and co-workers [76] studied the tautomeric equilibrium between **A** and **B** using semiempirical calculations (AM1 and PM3) and DFT (B3LYP/6-31G(d)) in the gas phase and water, where it was observed that in the gas phase **B** is the most stable, while in water **A** is formed, which is in agreement with the experimental results reported in the literature. There was no effect of the substituents on the phenyl group on the stability of the two tautomers.

**Scheme 4:**
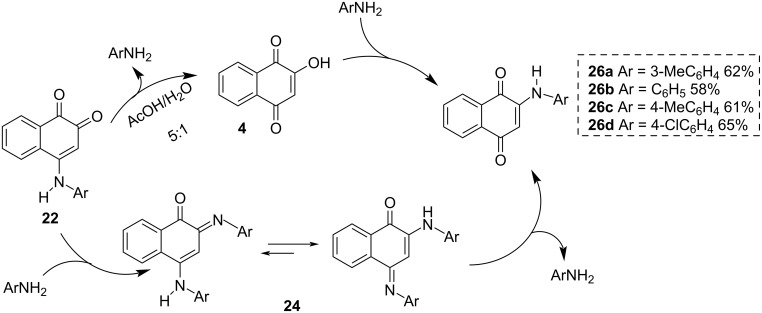
Isomerization of 4-arylamino-1,2-naphthoquinones.

A method to isomerize **22** to **26** was developed by Gornostaev and co-workers [77]. This method involves refluxing **22** in 85% aqueous acetic acid leading to **26** in 58–65% yield. The proposal to explain the isomerization involves two routes: one through the hydrolysis of **22** leading to lawsone (**4**) and the subsequent addition of arylamines in position C2, and the other involves the addition of arylamines at position C2 of **22**, leading to **24** with two equivalents of arylamine, which after hydrolysis of the imine at position C4 provides **26** (Scheme 4).

**Scheme 5:**
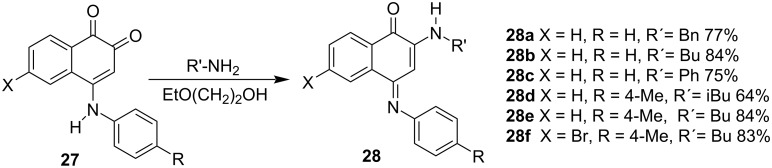
Synthesis of unsymmetrical 2-amino-4-imino compounds.

The same group developed reaction conditions using different hydrophilic solvents to synthesize unsymmetrical 2-amino-4-imino compounds **28** from **27** in good yields by employing primary aromatic amines (Scheme 5) [78].

**Scheme 6:**
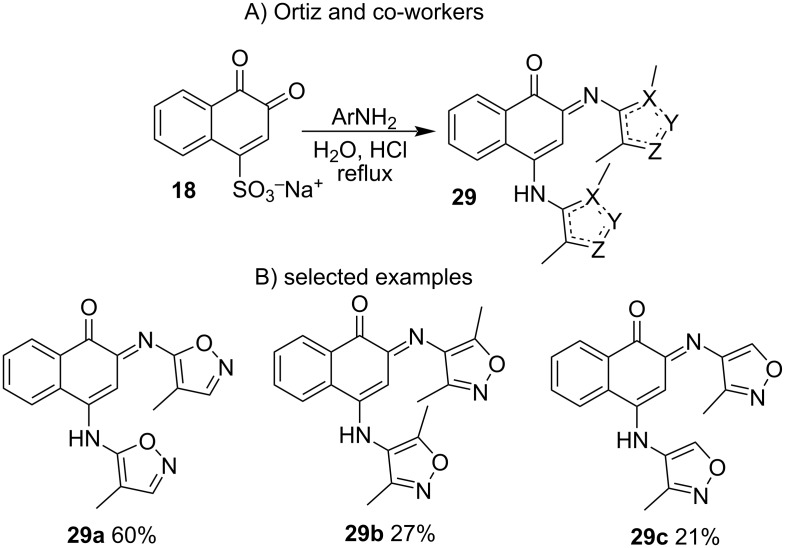
Synthesis of bis(isoxazolyl)naphthoquinones from β-NQS.

Ortiz and co-workers [79] performed several reactions employing two equivalents of isoxazolylamines in aqueous solution under reflux with HCl catalysis, resulting in bis(isoxazolyl)naphthoquinones **29** (Scheme 6).

The development of synthetic methodologies for the preparation of many bioactive substances is still a challenge. The main issues in synthetic organic chemistry and medicinal chemistry of naphthoquinones are the diversification of the strategies to obtain derivatives [8,80]. β-NQS are excellent electrophiles and have been used for obtaining naphthoquinones substituted by alkyl- or arylamines.

**Scheme 7:**
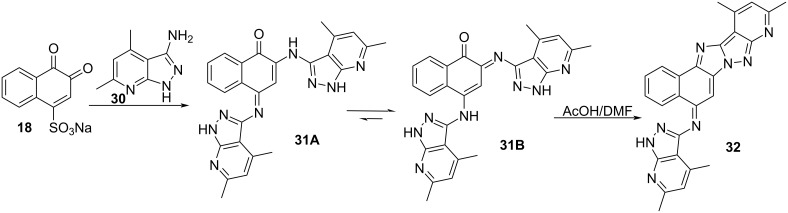
The reaction of β-NQS with **30** followed by cycle condensation.

The reactivity of aminopyrazolopyridine **30** with β-NQSNa (**18**) for the preparation of **32** with antioxidant properties was investigated by Gouda in 2012 [81]. Intermediate **18** was treated with two equivalents of **30** resulting in bispyrazolopyridine **31**, which after treatment with refluxing acetic acid produced imidazopyrazole **32** (Scheme 7). Compounds **31** and **32** were evaluated against their antioxidant activity and exhibited promising activity.

**Scheme 8:**
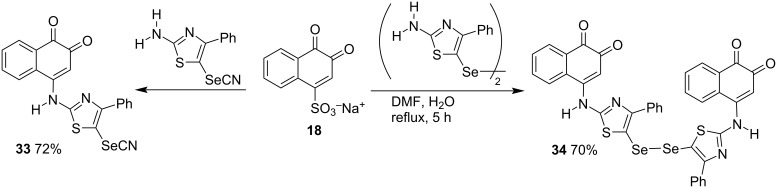
Synthesis of 4-(2-amino-5-selenothiazoles)-1,2-naphthoquinones.

Protein tyrosine phosphatase 1B (PTP1B) is essential in the dephosphorylation of the activated insulin receptor, and inhibition of this enzyme would be an excellent strategy for the treatment of type 2 diabetes. Ahn and co-workers [82] synthesized and evaluated several 1,2-naphthoquinones substituted at position C4 with alkyl- or arylamino groups for their inhibition of the PTP1B protein. Furthermore, to discover new effective anti-inflammatory and analgesic agents, Gouda and co-workers [83] synthesized various compounds in good yields from the reaction of β-NQS **18** with 2-amino-5-selenothiazoles, such as **33** and **34**. The authors reported that most of the compounds tested had similar anti-inflammatory properties or greater activity than meloxicam (Scheme 8).

**Scheme 9:**
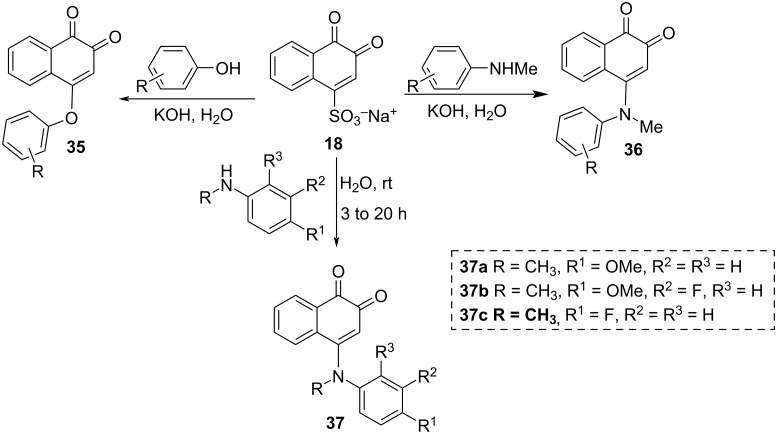
Synthesis of amino- and phenoxy-1,2-naphthoquinones.

β-Lapachone (**11**) is a potent reversible inhibitor of the liver enzyme human carboxylesterase (hCE1) that cleaves carboxylic esters. This enzyme functions in the detoxification metabolism of carcinogenic and mutagenic organic compounds, converting them into nontoxic metabolites. This compound served as inspiration for Hatfield and co-workers [84], who proposed the synthesis of a series of amino-*N*-methylated compounds **36** and phenoxy-1,2-naphthoquinones **35** with a carbon skeleton similar to β-lapachone (**11**), which could modulate hCE1 activity. Studies have shown that amino-*N*-methylated-1,2-naphthoquinones **36** are more selective and potent inhibitors than phenoxy-1,2-naphthoquinones **35** and β-lapachone (**11**) for hCE1 (Scheme 9). It is important to note that **35** can be obtained from other reagents, such as 2-naphthol, in a cascade of reactions involving oxidation to 1,2-naphthoquinones followed by Michael addition to the olefin and reoxidation [85]. In addition, Yang and co-workers synthesized other naphthoquinone derivatives **37** from β-NQSNa (**18**) [86]. These compounds were evaluated for their antiproliferative activities on human cancer cells, with three of them being the most active (**37a–c**). It has been shown that the mechanism of action passes through the production of intracellular ROS and includes inhibition of tubulin polymerization (Scheme 9).

**Scheme 10:**
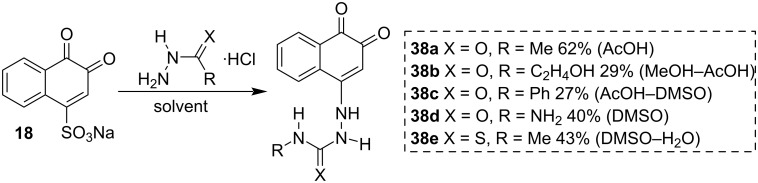
Synthesis of 4-semicarbazide-1,2-naphthoquinone.

Semicarbazides and thiosemicarbazides are substances used to identify aldehydes and ketones, are very versatile in the synthesis of heterocycles, and have several applications in the preparation of important drugs. Nucleophilic nitrogen semicarbazides can easily add to carbon C4 of β-NQSNa (**18**) to produce 1,2-naphthoquinones containing these groups [87]. This is a reaction very similar to adding amines to **18**. Yamada and co-workers [88] studied the preparation of several 4-semicarbazide- and 4-thiosemicarbazide-1,2-naphthoquinones by the reaction of **18** with semicarbazides and thiosemicarbazides to obtain new compounds with improved hemostatic activities. These compounds were obtained in moderate yields and were capable of reducing the bleeding time (Scheme 10).

### Synthesis of 4-azido-β-naphthoquinones from β-NQS

**Scheme 11:**
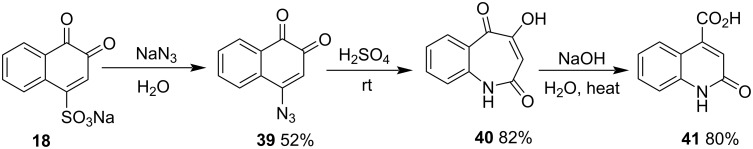
Reactions of 4-azido-1,2-naphthoquinone.

β-NQS can react with other nitrogenous nucleophiles, such as azide ions. The reaction of **18** in the presence of sodium azide and water produces 4-azido-1,2-naphthoquinone (**39**) in a 52% yield. Although **39** can produce many other derivatives, few reactions have been studied. The reaction of **39** with concentrated sulfuric acid at room temperature produces azepinedione **40** in an 82% yield. This compound can be transformed into several 3-substituted and 4-hydroxy derivatives. However, an interesting transformation results from the treatment of **40** with hot aqueous sodium hydroxide, resulting in 2-oxoquinoline **41** in an 80% yield (Scheme 11) [89].

### Modifications in β-carbonyls

**Figure 4:**
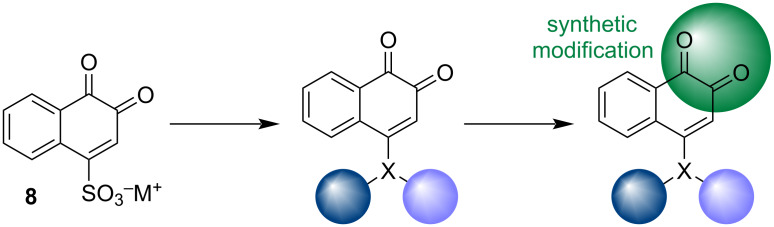
Modifications that can be easily carried out from the products of β-NQS **8**.

1,2-Naphthoquinone derivatives can be obtained through modifications in the carbonyls, leading to the formation of new compounds containing different groups, such as hydroxylamines, oxiranes, hydrazones, and heterocycles. These modifications can be easily carried out from the products of β-NQS **8** reactions with substituted amines and phenols (Figure 4). The objective of these transformations is to search for new compounds that present new physicochemical and biological properties.

**Scheme 12:**
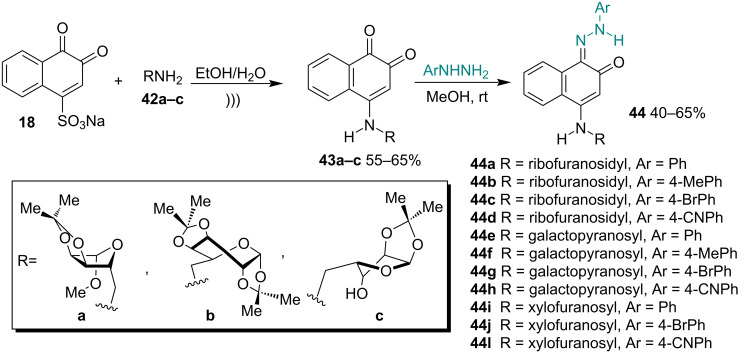
Derivatives of 1,2-naphthoquinones obtained from β-NQS.

For the first time, Campos and co-workers [90] performed the synthesis of novel 4-amino-1,2-naphthoquinones **43a–c** containing carbohydrates and evaluated their antitumor activity in vitro. These compounds were transformed into a series of hydrazones **44a–l** with different substituted aryl groups, which were also evaluated against tumor cells. To prepare **43a–c**, **18** was reacted with different amines **42a–c** in the presence of an ethanol–water mixture under ultrasonication, followed by reaction with arylhydrazines**,** which led to hydrazones **44a–l** according to the classical procedure that employs methanol at room temperature. All compounds were evaluated against different human cancer cell lines, including leukemia (HL-60), melanoma (MDA-MB-435), colon cancer (HCT-116), and central nervous system cancer (SF-295). 4-Amino-1,2-naphthoquinones **43a–c** exhibited considerable cytotoxic activities, with **43a** being the most active against HL-60 and MDA-MB-435 cells (Scheme 12).

**Scheme 13:**
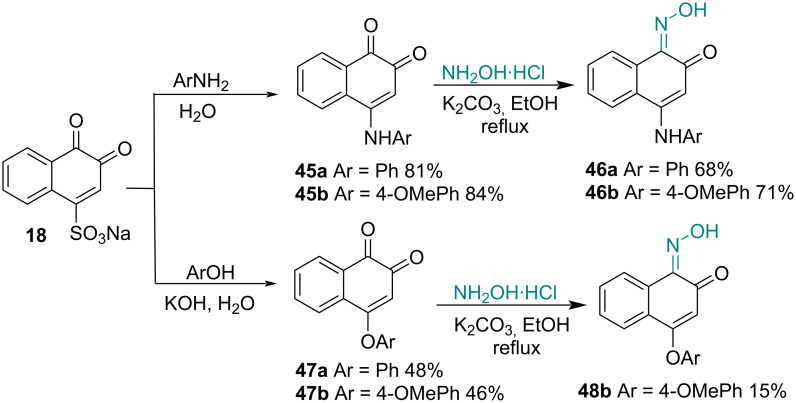
Oximes as well as 4-amino- and 4-phenoxy-1,2-naphthoquinone as potential anti-inflammatory agents.

Other naphthoquinone derivatives were synthesized with modifications at one of the carbonyls. Tseng and co-workers [91] synthesized 4-arylamino-1,2-naphthoquinones **45a**,**b** and 4-phenoxy-1,2-naphthoquinones **47a**,**b** as potential anti-inflammatory agents capable of inhibiting the expression of nitric oxide (NO) and PGE2 in alveolar macrophages. Then, oximes **46a,b** and **48b** were obtained by condensation of **45a,b** and **47b** with hydroxylamine, respectively. Biological results indicated that **47b** significantly attenuated the release of inflammatory mediators (NO, TNF-α, and MMP-9) in a concentration-dependent manner. These data indicated that **47b** targets p38 kinase and NF-κB and may serve as an anti-inflammatory agent (Scheme 13).

**Scheme 14:**
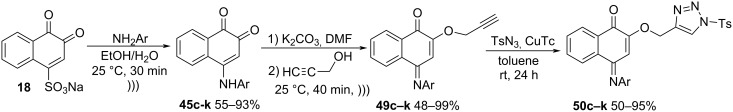
Synthesis of triazoles from β-NQS.

In 2020, Almeida and co-workers [92] synthesized naphthoquinone imines from β-NQSNa (**18**) with modifications in β-carbonyls. These compounds were obtained in a sequence of reactions involving the addition of arylamines to β-NQS **18** followed by *N*-alkynylation and then Cu(I)-catalyzed heterocyclization with tosyl azide in toluene at room temperature, leading to triazoles **50c–k** in moderate to excellent yields (Scheme 14).

**Scheme 15:**
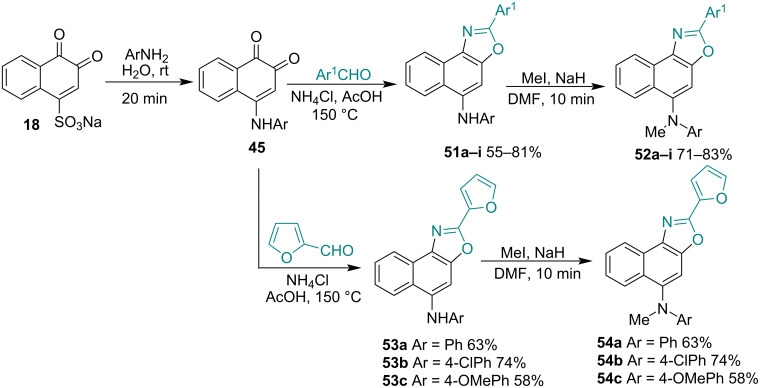
Synthesis of naphtho[1,2-*d*]oxazoles from β-NQS.

The research group that most explored the formation of heterocycles from 1,2-naphthoquinones was Pinto’s group, which prepared several imidazolyl, oxazolyl, phenoxazinyl, and indolyl heterocycles that were evaluated against some pharmacological targets [93–96]. Many of the methods that are in use today have been developed by this group. Lee and co-workers [97] developed the synthesis of naphtho[1,2-*d*]oxazole heterocycles from β-NQS as potential antiviral agents capable of inhibiting the HCV virus. Compound **45** was obtained from β-NQSNa (**18**) as shown above and reacted with substituted benzaldehyde or furfuryl aldehyde to form naphthoxazoles **51a–i** and **53a–c**, respectively. These compounds were then *N*-methylated, leading to the corresponding compounds **52a–i** and **54a–c**. Naphthoxazol **53c** was the most effective anti-HCV agent, exhibiting an IC_50_ value of 0.63 μM, higher than that of the standard drug (ribavirin, IC_50_ 13 μM) (Scheme 15). Naphthoxazoles stand out for exhibiting solid-state fluorescence, although the fluorescence partially disappears in solution, and there is a large shift to red and blue [98–99].

### Carbon–carbon bond formation

The main steps in a synthesis usually involve C–C bond formation, which is usually the main reaction step, or functional group transformations. Organometallics are the most commonly used catalysts to promote C–C bond formation. In addition, other so-called classical reactions are also widely used, such as Friedel–Crafts alkylation and acylation, Wittig and Horner–Emmons reactions, carbonyl addition/substitution, α-alkylation, aldol reactions, and pericyclic reactions.

**Scheme 16:**
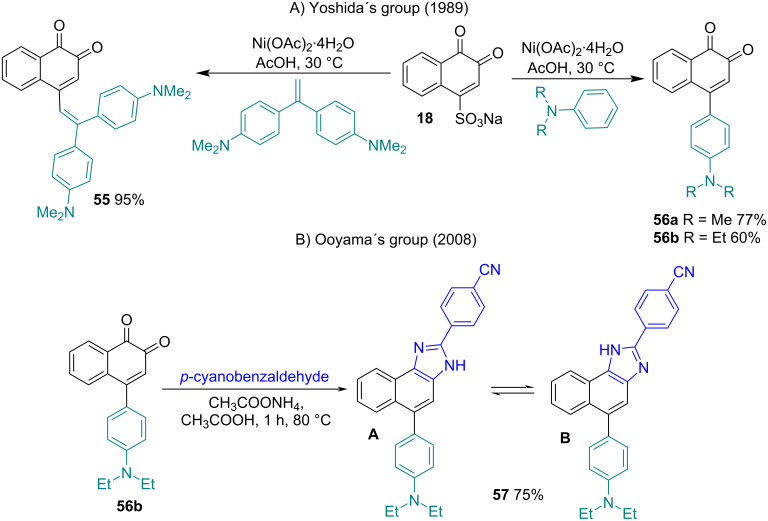
A) Arylation and vinylation of β-NQS catalyzed by Ni(II) salts. B) Transformation of the 1,2-dicarbonyl group in the fused imidazo[4,5-*a*] heterocycle.

Yoshida and co-workers [100] demonstrated that some metal ions are capable of activating aromatic compounds by chelation and promoting nucleophilic additions. For instance, 1-aminoanthraquinone quickly reacts with butylamine under the influence of Lewis acid catalysts to give 1-amino-4-butylaminoanthraquinone. Similarly, quinoline-5,8-diones react with amines under catalysis with Ni(II) ions to selectively give substituted amino derivatives [101–102]. The same group demonstrated that the reactions between β-NQS **18** and *N,N’*-dialkylanilines or 1,1-bis[*p*(dimethylamino)phenyl]ethylene in acetic acid efficiently produced 4-vinyl-1,2-naphthoquinones **55** and 4-aryl-1,2-naphthoquinones **56a,b**, respectively, under nickel(II) catalysis, forming a C–C bond (Scheme 16) [103]. When the reaction was carried out in 10% aqueous methanol solution at room temperature for 5 hours, **56b** was produced in an 85% yield [104–105]. Then, Ooyama and co-workers [99] transformed the 1,2-dicarbonyl group into the fused imidazo[4,5-*a*] heterocycle via a reaction of **56b** with 4-cyanobenzaldehyde and an NH_3_ source in a 75% yield. The crystal of **57** exhibits a sensitive color change and fluorescence enhancement behavior with a blueshift in the emission maximum upon enclathration of various types of organic solvents. It is important to note that other authors carried out the reaction of **18** with *N,N*-diethylaniline without the presence of Ni(II) [104–105].

**Scheme 17:**
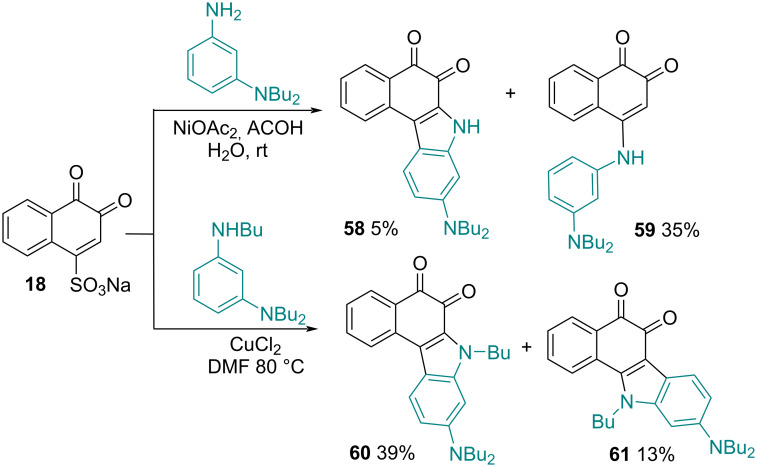
Benzo[*a*]carbazole and benzo[*c*]carbazoles fused with 1,2-naphthoquinone.

In the search for fluorophores of heterocyclic quinoid type to study their photophysical properties in solution and in the solid-state, Ooyama and co-workers [106] studied a synthetic route for the preparation of compounds with the tricyclic benzo[*c*]carbazol-6-one skeleton. The strategy used was through the reaction to β-NQS **18** with a bifunctional amine (3-amino-*N,N*-dibutylaniline) under NiOAc_2_ catalysis to obtain the 4-arylated compound. As expected, 4-arylated-benzo[*c*]carbazole-5,6-dione **58** and 4-amino-1,2-naphthoquinone **59** were formed in 5% and 35% yields, respectively. However, the reaction of **18** with 3-butylamino-*N,N*-dibutylaniline in DMF in the presence of CuCl_2_ formed two isomers of 4-arylated-*N*-butylbenzo[*c*]carbazole-5,6-dione **60** and 4-amino-benzo[*a*]carbazol-5,6-dione **61** in 39% and 13% yields, respectively. These two reactions demonstrate the importance of the catalyst in complex formation with carbonyls of **18** that promote nucleophilic desulfoamination or nucleophilic desulfoarylation at position C4, and the following intramolecular cyclization occurs to produce 1,2-naphthoquinones fused with the benzo[*a*]carbazole or benzo[*c*]carbazole system (Scheme 17).

**Scheme 18:**
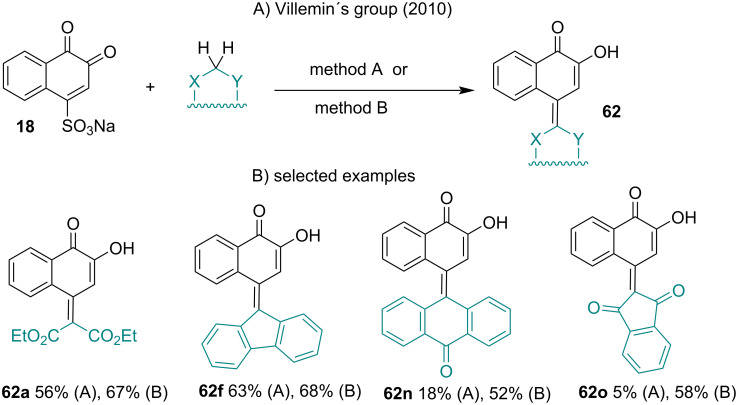
Synthesis of 1,2-naphthoquinones having a C=C bond from β-NQS. Method A: NaOH, EtOH/H_2_O, 40 °C, 2 h; Method B: *t-*BuOK, PEG-300, rt, 6–10 min.

An interesting reaction for the formation of 4-cyanoethyl-l,2-naphthoquinone from β-NQS^−^M^+^ was developed by Gates and Newhall in 1948 [107]. Land and co-workers demonstrated that cyanomethyl derivatives of *ortho*-quinones undergo facile tautomerism to *para*-quinomethanes [108]. Villemin and co-workers summarized these reactions, which were expanded to several other condensation products with methylene acid compounds [109]. These authors developed two methods (A and B) to prepare 2-hydroxynaphthoquinomethanes **62** with diverse structures by condensation of **18** with active methylene compounds (Scheme 18). Method A involves the reaction promoted by sodium hydroxide in an ethanol/water mixture at 40 °C, and method B was carried out with *t-*BuOK in polyethylene glycol (PEG300) at room temperature. In both methods, the reaction conditions were mild and produced the product in moderate to good yields.

**Scheme 19:**
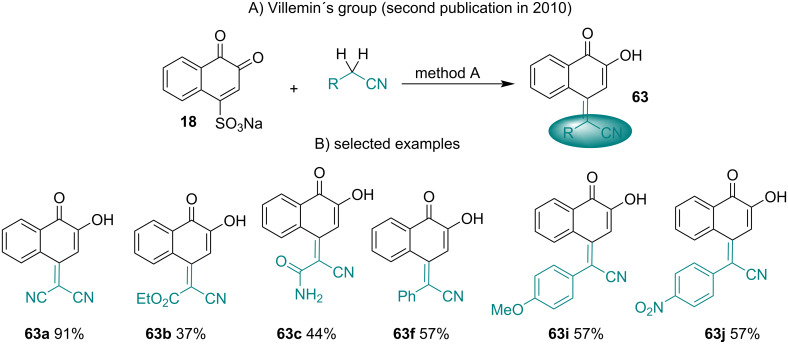
C=C bond formation from β-NQS and substituted acetonitriles.

The same group investigated this reaction with substituted acetonitriles to obtain 2-hydroxynaphthoquinomethanes [110]. These reactions were carried out by method A described above, and the stereochemistry was attributed to the *E*-isomer (Scheme 19).

## Conclusion

Compounds containing the 1,2-naphthoquinone scaffold represent a class of natural and synthetic substances with important biological activities and are therefore relevant for studies in the field of medicinal chemistry. The salts of 1,2-naphthoquinone-4-sulfonic acid (NQS) are the reagents of choice for performing selective transformations at position C-4 and preparing new 1,2-naphthoquinones with promising pharmacological properties. β-NQS is also important in quantitative analytical determinations of drugs containing free primary and secondary amino groups, as they react quickly and in high yield with amines to form colored products in good yields.

This review reports several examples of syntheses of functionalized 1,2-naphthoquinones substituted at the C4 position of β-NQS. Despite great advances in the area, there are still many opportunities for the development of new bioactive compounds of great relevance to humanity. We hope this article will serve as a source of inspiration for current and future researchers in chemical, pharmaceutical, and biological sciences.

A lot of the literature in this area is quite old. Perhaps there are also opportunities in this area to apply modern chemistry methodology, and also in the development of more sophisticated sensors and dyes.
